# Is Higher Nurse Aide Retention Associated with Fewer Nursing Home Allegations and Complaints?

**DOI:** 10.1093/geroni/igab046.3086

**Published:** 2021-12-17

**Authors:** Katherine Kennedy

**Affiliations:** Miami University, Oxford, Ohio, United States

## Abstract

Consumer voices are often left out from assessments of nursing home (NH) quality. For this reason, consumer allegations and complaints against nursing homes were studied in relation to facility rates of nurse aide retention. Analyses involved means and frequencies, correlations, ANOVAs with Tukey correction to examine the independent and dependent variables (N=690). Four quartiles of retention were created. In the final models, medium, high, and extremely high retention facilities are compared to the low retention facilities. Negative binomial regressions were estimated on total, substantiated, and unsubstantiated counts of allegations and complaints. All regressions controlled for the same characteristics, including nurse aide empowerment, consistent assignment, administrator turnover, director of nursing turnover, average age of residents, and percent female. The correlation between retention and the dependent variables was negative and statistically significant (r=-0.11, p<.01). The ANOVAs showed that high retention NHs (61-72%) received significantly fewer allegations than low (0-48%) and medium (49-60%) retention NHs; they also received fewer unsubstantiated allegations, and fewer complaints, both substantiated and unsubstantiated. After controlling for other variables, each retention group was significantly related to having fewer allegations and complaints compared to the low retention NHs. Notably, high retention NHs received between 29 and 35% fewer allegations and complaints of all types. Unexpectedly, extremely high retention NHs had more allegations, complaints, and unsubstantiated allegations than high retention NHs. Policy and practice have a role to promote nurse aide retention, improve job quality, and ensure adequate support for this critical, in-demand workforce.

